# Impact of Lighting Intensity on Welfare and Performance in Broiler Chickens

**DOI:** 10.3390/ani15223348

**Published:** 2025-11-20

**Authors:** Shengyu Zhou, Tanner Thornton, Hao Gan, Tom Tabler, Yang Zhao

**Affiliations:** 1 Department of Animal Science, The University of Tennessee, Knoxville, TN 37996, USA; szhou21@vols.utk.edu (S.Z.); tthorn15@vols.utk.edu (T.T.); gtabler@utk.edu (T.T.); 2 Department of Biosystems Engineering & Soils Science, The University of Tennessee, Knoxville, TN 37996, USA; hgan1@utk.edu

**Keywords:** broiler, lighting intensity, production performance, welfare

## Abstract

**Simple Summary:**

Lighting intensity (LI) plays a key role in how chickens grow and behave. In this study, we ran two separate trials to see how different LIs affect broilers. Trial 1 (July 2023) raised Ross 708 birds, and Trial 2 (October 2023) raised Cobb 700 birds. Each group was raised for eight weeks under three LIs (50, 20, and 5 lux). We tracked their growth, walking ability, feather condition, body temperature, and bone strength. We found that LI affected both growth and welfare, but not always in the same way for the two strains. Ross broilers grew slightly slower under 50 lux on day 42, while Cobb broilers were less efficient at converting feed into weight under 5 lux. Birds in bright light tended to walk better, while those in dim light had better feather coverage and lower surfaces temperature and 20 lux lighting sometimes led to dirtier feathers. These findings suggest that LI has different effects depending on the broiler strain and age. Providing appropriate LI can help optimize bird health, energy use, and production outcomes on commercial farms.

**Abstract:**

Lighting intensity (LI) affects broilers’ behavior, circadian rhythms, physiology, and welfare, making it essential in commercial broiler management. This study evaluated the effects of three LIs (50, 20, and 5 lux) on Ross 708 and Cobb 700 broilers from day 8 to 56 in separate trials conducted under identical conditions. Growth performance (body weight (BW), feed intake, feed conversion ratio (FCR)) and welfare indicators (gait, feather condition, temperature, and footpad dermatitis were assessed biweekly. Ross broilers at 50 lux showed reduced BW on day 42 (*p* = 0.03), with no BW differences observed on day 56 (*p* = 0.14). FCR was unaffected by LI for Ross broilers. For Cobb broilers, BW was not affected by LI, though birds under 5 lux exhibited approximately 2.6% lower FCR on day 28 (*p* = 0.04) and 7.8% lower FCR on day 42 (*p* = 0.01). LI of 50 lux was associated with increased back temperatures, while bird at 5 lux showed better feather coverage and lower belly temperature (*p* < 0.01). Ross under 20 lux showed poorer feather cleanliness (*p* < 0.01), while those under 50 lux had better gait scores on day 56 (*p* < 0.01). These results demonstrate that production and welfare responded differently to LI, and that these responses also varied between strains. These findings contribute to sustainable poultry production by balancing welfare, efficiency, and growth.

## 1. Introduction

In 2024, the United States produced 28.1 billion kg of broilers, representing approximately a 50% increase in broiler production since 2000 and surpassing swine and cattle production [1]. Such increased production in the broiler industry has made high-quality chicken meat more affordable. However, this rapid growth has been accompanied by rising public concern regarding the welfare of birds raised in intensive systems, particularly those kept under crowded and dimly lit conditions that restrict movement and natural behaviors [2,3].

Animal welfare originally defined by the “five freedoms” framework [4], later refined by Mellor [5], has evolved to emphasize both the prevention of negative states and the promotion of positive experiences. Accordingly, broiler producers are under increasing pressure to balance efficiency with welfare expectations from consumers and certification programs [6].

Among environmental factors, lighting, including intensity, photoperiod, and wavelength, plays a central role in shaping broiler physiology, behavior, and overall welfare [7]. In particular, light intensity (LI) directly influences activity levels, resting behavior, and welfare outcomes, yet the impact of LI on broiler production performance remains a subject of debate [7]. Some studies have reported that broilers reared in lower LI (5 lux) achieved higher body weight (BW) [8], and better feed conversion ratios (FCR) [9] compared to those exposed to 20 lux LI. These benefits are often attributed to reduced activity levels, which enable more efficient energy utilization [10]. Lower LIs are also associated with reduced activity and aggression [11,12], potentially contributing to improved production efficiency. However, other studies have found no significant effect of LI on broiler growth performance, suggesting that under certain management or environmental conditions, broilers may adapt to a range of LIs without compromising productivity [7,13,14,15,16,17,18,19].

Despite potential production benefits, prolonged exposure to dim LI has raised considerable welfare concerns. These include abnormal ocular development such as enlarged eye size and increased susceptibility to eye disorders [7,20], higher carcass fat content [21], leg problems [22] and potential disruption of vision and natural behavioral rhythms, ultimately compromising welfare [20]. Broilers exposed to dim lighting (5 lx) experienced a less distinct light-dark cycle, leading to reduced activity during the light phase and increased activity during the dark phase. In contrast, higher intensities (50 and 200 lx) promoted clearer circadian rhythms with activity peaks around light transitions [21]. Brighter environments also promote natural behaviors such as preening and walking, which serve as important indicators of broiler comfort and welfare [23].

Current recommendations LIs in the US vary. Broiler producers typically use 5–10 lux after brooding to improve the growth rate and feed efficiency [24], while animal welfare advocates and food chain companies demand 20–50 lux throughout the entire production cycle [25]. Additionally, while many previous studies have focused on a single broiler strain, such as Ross [9,13,15] or Cobb [8,20], our study included both Ross and Cobb strains, together representing approximately 90% of the commercial broiler market in the U.S. This broader strain coverage enhances the applicability of our findings and highlights the importance of considering strain-specific responses when evaluating the effects of LI.

The objective of this study was to comparatively evaluate the production performance and welfare of Ross 708 and Cobb 700 broilers as affected by LI. Specifically, we looked at BW, FCR, and a range of welfare indicators, including feather coverage, cleanliness, footpad, gait score and bone strength. Among these, feather coverage was quantitatively evaluated using a precision livestock farming (PLF) approach.

## 2. Materials and Methods

### 2.1. Birds, Diets, and Management

A total of 324 Ross 708 and 324 Cobb 700 straight-run broilers were obtained from local commercial hatcheries and reared at a 44 kg/m^2^ stocking density (18 birds per pen, pen size: 1.1 m × 1.5 m) until day 56. The selected density aligns with the National Chicken Council (NCC) Broiler Welfare Guidelines, which recommend up to 44 kg/m^2^ for market-weight broilers, considering the space required for birds to express normal behaviors [24]. The Ross trial was conducted in July 2023, followed by the Cobb trial in October 2023. All birds were reared under identical room conditions but housed separately in pens. Ross chicks were sexed by feather pattern, whereas Cobb chicks were sexed by vent examination, and both were assigned to pens to achieve an equal number of males and females. Each pen had a 36 cm tube feeder and a drinker line with three nipples, meeting NCC recommendations [24].

The tested LIs were 50, 20, and 5 lux applied from day 8 to day 56. Each LI treatment has 6 replicates. During the first week, all treatments were exposed to 50 lux lighting to help the chicks locate feed and water efficiently. All birds were reared in one room, with areas of varying LIs distinguished by black waterproof plastic tarps (Manufacturer: TEZONG; country of origin not specified, 10 × 20 ft each). To achieve lighting levels of 50, 20, and 5 Lux, 6, 4, and 3 light bulbs (Overdrive A19 Dimmable Omni LED Bulb—9.8 Watts 5000 K) were used, respectively, to provide lighting. To ensure even distribution of light, bulbs were spaced uniformly, and black tapes (Lichamp black electrical tape) were applied to the bulbs to fine-tune the lighting levels. LI at the bird level was measured using a HATO ONE spectrometer (Item code: 6776, HATO BV, Sittard, The Netherlands). Measurements were taken at three points in each pen: the right-up corner, center area, and left-down corner.

Room temperatures were regulated by age in accordance with the Ross 708 [26] and Cobb 700 [27] management guidelines. At trial onset, each pen was prepared with 0.14 ft^3^ of topsoil (Farmer Green, Garick LLC, Cleveland, OH, USA) overlaid with 0.04 ft^3^ of black shredded mulch (Earthgro, The Scotts Miracle-Gro Company, Marysville, OH, USA), and this bedding was maintained for the entire experiment. Topsoil and mulch provided a dark background that improved contrast with the white birds and enabled more accurate video-based monitoring and image analysis of broiler activities.

Feed was added daily, and the amount offered to each pen was recorded using a pre-weighed bucket. Biweekly FI was then calculated by subtracting the remaining feed from the total feed added over that period. At the same two-week intervals, the combined BW of all birds in each pen was measured, and these data were used to calculate FCR (feed intake kg/body weight gain kg). Broilers were reared to a target market weight of 4.0 kg for 56 days. Broilers in this study were provided with a diet containing 19% crude protein and 2851 kcal/kg metabolizable energy (Co-op Chick Starter/Grower Crumble—AMP BMD, Tennessee Farmers Cooperative, La Vergne, TN, USA) throughout the entire study.

### 2.2. Welfare and Behavior Measurement

Broiler welfare was evaluated on days 28 and 56 by evaluating feather cleanliness, footpad condition, and gait score following procedures adapted from Zhou et al. [28] and the Welfare Quality^®^ assessment protocol [29]. On each assessment day, two males and two females were randomly sampled from each pen. The selected birds were briefly removed from their pens for individual scoring.

Each welfare indicator was rated on a standardized scale, where lower scores indicated better welfare status. Feather cleanliness was scored from 0 (clean) to 3 (heavily soiled), footpad lesions were rated from 0 (no visible damage) to 4 (severe ulceration), and gait was assessed on a 0–5 scale, with scores of 3 or higher considered indicative of impaired mobility and compromised welfare [30].

As part of PLF, thermal imaging and image analysis were employed to assess body surface temperature and bare-skin exposure. The thermal camera (T865, Teledyne FLIR, Wilsonville, OR, USA) captured surface temperatures of the back and belly regions, which correspond to the back and belly areas as defined by Zhao et al. [31]. These measurements were then used to calculate the bare skin ratio. Bare skin ratio calculations used a threshold of 33.5 °C on day 28 and 35 °C on day 56 for both strains.

At the end of the flock, bone strength was assessed in two randomly selected birds (one male and one female, euthanized via carbon dioxide method) from each pen. Breaking strength of the left tibia was assessed via a three-point bending procedure on an MTS Alliance RT/30 instrument (MTS Systems Corporation, Eden Prairie, MN, USA), with supports positioned 4 cm apart. All experimental procedures were conducted in accordance with the guide for the Care and Use of Agriculture Animals in Research and Teaching [32], and approved by the Institutional Animal Care and Use Committee (IACUC Protocol #2876-1221).

### 2.3. Statistical Analysis

All statistical analyses were performed in JMP (version 16.0.0, SAS Institute, Cary, NC, USA). Treatments of 50, 20, and 5 lux were used in a complete randomized design with six replications. Welfare-related traits: feather coverage, feather cleanliness, bone strength, footpad dermatitis, and gait score were analyzed at the individual-bird level. Because birds were sampled at different ages, those evaluated on day 28 were not necessarily the same individuals assessed on day 56. The pen was regarded as the experimental unit for the weight gain and FCR. The Mixed Analysis of Variance (ANOVA) was used to test the effects of LI on continuous variables (bone breaking strength, average temperature, and bare skin ratio), with pen included as a random blocking factor. Scored variables (gait score, footpad dermatitis, feather coverage, and feather cleanliness) were examined using a Mixed ANOVA on ranks to assess the effect of LI. Tukey’s HSD test was applied for post hoc pairwise comparisons, with significance set at the 0.05 level, following the calculation of least squares means. Residual normality was evaluated via the Shapiro–Wilk normality test. Additionally, Kruskal–Wallis test was used to compare day 28 and day 56 measurements for both scored and continuous traits, with significance declared at *p* < 0.05.

## 3. Results

### 3.1. Effect of Lighting Intensity on Body Weight and FCR

According to Table 1, LI had a significant effect on the BW of Ross 708 broilers on Day 42 (*p* = 0.03), where birds at 20 lux had the highest average weight (2.70 kg), followed by those under 5 lux (2.61 kg) and 50 lux (2.54 kg). However, no significant differences in BW were detected on Days 14, 28, or 56 (*p* > 0.05), although the 5 lux group consistently showed slightly higher weights at most time points. LI did not significantly affect FCR at any age for the Ross bird (*p* > 0.05).

For Cobb 700 broilers, BW was not significantly influenced by LI at any stage of growth (*p* > 0.05), although there was a numerical trend toward higher final weights in the 5 lux group on Day 56 (4.28 kg) compared to the 20 lux group (4.03 kg). In contrast, FCR was significantly affected by LI on Days 28 and 42. On Day 28 (*p* = 0.04), birds at 20 lux and 5 lux had lower FCR values (1.52 and 1.51, respectively) than those at 50 lux (1.55). Similarly, on Day 42 (*p* = 0.01), the lowest FCR was recorded under 5 lux (1.66), while the highest was under 20 lux (1.80). No differences in FCR were observed on Days 14 or 56.

### 3.2. Effect of Lighting Intensity on Bone Breaking Strength

According to Table 2 and Table 3, LI does not affect the bone breaking strength in either Ross or Cobb broilers. However, within both strains, male broilers exhibit significantly higher bone strength than females, with male bone strength approximately 1.25 times that of females.

### 3.3. Effect of Lighting Intensity and Age on Body Temperature and Bare Skin Ratio

As shown in Table 4, on day 28, Ross broilers exposed to 50 lux had significantly higher back temperatures (33.08 °C) than those under 20 lux (31.51 °C), with a difference of 1.57 °C (*p* = 0.03). This increase may result from higher activity levels under higher LI, as greater exercise and competition can reduce back feather coverage. In addition, back temperatures on day 28 were higher than those on day 56 across all LIs. As broilers matured, feather coverage on the back and abdomen increased, reducing exposed skin areas and reflecting normal feather growth and development patterns.

As shown in Table 5, Cobb broilers on day 56 exposed to 5 lux had a lower bare skin ratio on the belly than those under 20 or 50 lux. The better feather coverage lowered belly temperature by 1 °C compared to higher light groups. The improved coverage may be due to less friction with the litter, as lower LI can reduce movement and help maintain feather condition. Feather coverage on the back and abdomen also improves as broilers grow.

### 3.4. Effect of Lighting Intensity on Gait Score, Footpad and Feather Cleanliness

As shown in Table 6 and Table 7, LI influenced feather cleanliness and gait scores in Ross. On day 28, broilers under 20 lux showed the dirtiest feathers (1.07), while those under 50 lux had the best cleanliness score (0.80). On day 56, the 50 lux group had the best gait score (1.72), above the 20 lux (2.26) and 5 lux (2.25) groups.

For Cobb on day 56, LI affected both gait and footpad dermatitis scores. Birds at 50 lux showed better gait score (2.04), whereas the 5 lux group showed the best footpad dermatitis score (2.13), suggesting higher LI improves gait, while lower LI supports footpad health.

LI had no significant effect on the gait score on day 28 or footpad condition on both 28 and 56 days in Ross broilers. Similarly, LI did not influence the gait score or footpad condition of Cobb on day 28, nor feather cleanliness on both 28 and 56 days.

### 3.5. Effect of Age on Gait Score, Footpad and Feather Cleanliness

As shown in Table 8, age significantly affects all welfare indicators (all *p* < 0.01) in both Ross and Cobb broilers. Feather cleanliness declines with age, while gait scores and footpad health deteriorate as the birds gain weight in both strains.

## 4. Discussion

### 4.1. Effect of Lighting Intensity on Body Weight and Feed Conversion Ratio

In this study, we evaluated the effect of different LIs on broiler production performance. Our findings indicate that Ross 708 broilers exposed to 50 lux had lower BW on day 42 compared to those under 20 lux. A similar trend was observed in Ross 308 broilers on day 46, where birds under 20 lux (BW: 2.79 kg) showed lower BW than those under 5 lux (BW: 2.91 kg), likely due to increased activity and energy expenditure [9]. However, our results showed a different trend where BW of Ross under different LIs had no significant difference on day 56. Similarly, Olanrewaju et al. [33] reported that BW (4.14 kg) and FCR (2.10) of Ross 708 broiler on day 56 were unaffected by LIs ranging from 25 to 0.2. This suggests that LI may have an age-dependent effect on growth, with birds under higher LI potentially catching up in weight between days 42 and 56. Overall, our study showed that LI has age-dependent effect on the Rosses’ BW. In contrast, FCR in Ross broilers remained unaffected by LI throughout the trial. This is consistent with earlier studies [13,14,15] showing that LI variations do not significantly influence feed efficiency in broiler production.

For Cobb 700 birds, broilers’ FCR was affected by LI. Cobb particularly favor 5 lux LI. Broilers reared under 5 lux had a lower FCR on both day 28 (1.51 vs. 1.55 under 50 lux) and day 42 (1.66 vs. 1.80 under 20 lux), suggesting improved feed efficiency under dimmer conditions. However, their BW was unaffected by LI, with averages of 2.88 kg on day 42 and 4.16 kg on day 56. Aldridge et al. [8] reported a similar trend in Cobb 700 broilers, their BW (2.58 kg) on day 42 was unaffected by LI, and birds raised under 5 lux had better FCR (1.69) compared to those under 20 lux (1.76) on day 42, likely due to reduced activity and more efficient energy use. However, on day 56 in our study, the advantage in FCR under 5 lux LI had disappeared, with no significant differences observed among LI treatments. This suggests that the impact of LI on feed efficiency may be age-dependent, with stronger effects between days 28 and 42, and a diminished influence as birds approach market weight on day 56.

While dim light environment appears to improve BW or FCR at certain stages, excessively low LI (<1 lux) can have adverse effects. Arowolo et al. [34] found that very low LI significantly limited feed and water intake, leading to stunted growth and poor FCR. Moreover, prolonged exposure to LI below 5 lux could increase the risk of health issues such as eye abnormalities or footpad dermatitis [15,35]. Conversely, higher LI (e.g., ≥20 lux) during the first 7 days of age may promote exploration and facilitate access to feed and water, which is crucial during the first week of life [35]. This highlights the importance of adapting LI according to strain and stage. Pal et al. [12] recommended beginning with higher intensities (~20 lux) during the first week to stimulate early activity and intake, followed by a reduction to 3–5 lux to improve FCR and energy efficiency. Other studies have also linked lower LIs (e.g., 5–10 lux) with reduced hyperactivity, energy conservation, and improved growth and FCR outcomes [35,36].

Overall, the effect of LI on production performance was limited and strain-dependent. Ross broilers under 20 lux showed higher BW than those under 50 lux on day 42, while Cobb broilers reared under 5 lux exhibited better FCR on days 28 and 42. No significant differences in BW or FCR were observed at other ages.

### 4.2. Effect of Lighting Intensity on the Welfare of Broilers

Bone breaking strength of Ross or Cobb broilers was unaffected by LI, consistent with findings from previous research. Deep et al. [7] found no significant differences in skeletal scores or gait ability across different LIs, suggesting that LI alone does not directly influence skeletal development or walking ability. Similarly, Škrbić et al. [37] found that LI did not affect tibial weight, length, or cross-sectional area, across different stocking densities (10, 13, or 15 birds/m^2^).

LI significantly affected broiler surface temperatures in this study. For Ross broilers on day 28, back temperatures were 1.43 °C higher at 50 lux group compared to the 20 lux group. The elevated surface temperature in the 50 lux group may be attributed to more aggressive behavior, feather damage and loss, and thermal regulation triggered by higher LI. As Buyse et al. [38] reports, high LI can promote aggressive behaviors such as fighting, feather pecking, and cannibalism. Enhanced standing and walking under brighter LI aligns with findings from Deep et al. [7] and Wu et al. [35], who observed that higher LI encourages activity but may also increase risks of overstimulation and social stress. Cobb broilers reared under 5 lux on day 56 exhibited lower belly temperature and bare skin ratios compared to those under 20 lux and 50 lux. These suggest that broilers in 5 lux LI environments had better feather coverage, emphasizing the protective effect of 5 lux LI on feather condition. Improved feather coverage not only provides a physical barrier that reduces the risk of skin injuries but also reflects better overall welfare. Furthermore, lower LI effectively reduces aggressive behaviors such as feather pecking, also contributing to improved feather coverage and welfare outcomes [12,34]. LI of 5–10 lux may offer a balance between activity and rest, potentially reducing the risk of aggression and skin injuries [35].

Ross and Cobb broilers exposed to 20 lux had the least clean feathers on day 56. The increased litter moisture under 20 lux LI may have contributed to poorer feather cleanliness. Litter in the 20 lux group appeared visibly wetter compared to the 5 lux group during routine observations from approximately day 40, although litter quality was not systematically recorded, as it was not included in the initial experimental design. For Cobb broilers under 20 lux on day 56, more severe footpad dermatitis was also observed, likely in association with the wetter litter conditions. A similar trand was reported by Kang et al. [39] who found that broilers housed under 20 lux had wetter litter compared with those under natural and variable lighting. The authors suggested that reduced activity and poorer litter drying under constant 20 lux conditions contributed to the higher litter moisture. These findings together suggest that elevated light levels may compromise litter conditions, which in turn can impact broiler feather cleanliness and welfare. Additionally, considering that the waterproof black tarp was installed on the same side as the waterline, its use may have hindered pen ventilation and litter drying, further contributing to increased litter moisture. Both Ross and Cobb broilers raised under 50 lux exhibited better gait scores on day 56, which aligns with findings that higher LIs promote activity and improve leg health [37]. Greater activity under higher LIs reduced contact with wet litter, mitigating footpad issues. On day 28, gait scores, footpad conditions, and feather cleanliness were unaffected by LI in either strain.

Overall, these findings highlight that LI exerts multidimensional effects on broiler welfare, and no single level can achieve optimal outcomes across all traits. Thus, lighting management should be tailored to production goals, balancing gait score, footpad, and feather condition, to ensure sustainable performance and animal well-being.

## 5. Conclusions

In conclusion, for Ross broilers on day 42, exposure to 50 lux showed lower BW compared to other LIs, though this difference was no longer observed by day 56, and FCR remained consistent across all LIs. Conversely, Cobb broilers’ BW was unaffected by LI, but broilers at 5 lux showed a higher FCR on day 42. Bone strength was consistent across LIs for both strains, with males having approximately 1.25 times greater bone strength than females. LI influenced feather coverage, as Ross birds at 50 lux LI showed higher back temperature on day 28, while Cobb broilers under 5 lux on day 56 maintained better belly feather coverage and had lower belly temperatures. Broilers under 20 lux had poorer feather cleanliness, while those under 50 lux exhibited better gait scores on day 56 in both strains. Age significantly influenced welfare, with feather cleanliness, gait, and footpad health declining as broilers matured. Overall, LI had strain- and age-dependent effects on broiler welfare and performance. These findings provide practical implications for sustainable poultry production by supporting decisions that balance animal welfare, energy efficiency, and growth performance.

## Figures and Tables

**Table 1:** Effect of lighting intensity (LI, lux) on the body weight (BW, kg) and feed conversion ratio (FCR) of Ross 708 and Cobb 700 broilers.

**Ross 708**						
BW (kg) or FCR	50 lux	20 lux	5 lux	Average	Std Error	*p*-value
BW (Day 14)	0.34	0.35	0.35	0.35	0.01	0.10
BW (Day 28)	1.10	1.18	1.15	1.14	0.03	0.13
BW (Day 42)	2.54 ^b^	2.70 ^a^	2.61 ^ab^	2.62	0.04	0.03
BW (Day 56)	4.04	3.84	3.97	3.95	0.06	0.14
FCR (Day 14)	1.32	1.32	1.28	1.31	0.03	0.58
FCR (Day 28)	1.37	1.38	1.41	1.39	0.03	0.60
FCR (Day 42)	1.72	1.73	1.75	1.73	0.02	0.45
FCR (Day 56)	1.91	1.98	1.92	1.94	0.02	0.37
**Cobb 700**						
BW (kg) or FCR	50 lux	20 lux	5 lux	Average	Std Error	*p*-value
BW (Day 14)	0.44	0.44	0.45	0.44	0.01	0.76
BW (Day 28)	1.44	1.47	1.49	1.46	0.02	0.26
BW (Day 42)	2.88	2.79	2.98	2.88	0.06	0.13
BW (Day 56)	4.17	4.03	4.28	4.16	0.07	0.07
FCR (Day 14)	1.21	1.20	1.19	1.20	0.01	0.47
FCR (Day 28)	1.55 ^a^	1.52 ^ab^	1.51 ^b^	1.53	0.01	0.04
FCR (Day 42)	1.75 ^ab^	1.80 ^a^	1.66 ^b^	1.74	0.03	0.01
FCR (Day 56)	2.04	1.96	2.00	2.00	0.04	0.44

^a^, ^b^ Different superscript letters within a row indicate significant differences (*p* < 0.05).

**Table 2:** Effects of lighting intensity (LI, lux), and sex on Ross 708 broiler’s bone breaking strength (lbf).

**Effect of LI on Bone Strength of Ross**							
50 lux	20 lux		5 lux	Average	Std Error		*p*-value
122.30	119.00		140.19	127.16	7.43		0.11
**Effect of sex on bone strength of Ross**							
Male		Female		Std Error		*p*-value	
142.26 ^a^		112.06 ^b^		6.07		<0.01	

^a^, ^b^ Different superscript letters within a row indicate significant differences (*p* < 0.05).

**Table 3:** Effects of lighting intensity (LI, lux), and sex on Cobb 700 broiler’s bone breaking strength (lbf).

**Effect of LI on Bone Strength of Cobb**							
50 lux	20 lux		5 lux	Average	Std Error		*p*-value
103.17	107.58		111.65	107.47	7.19		0.71
**Effect of sex on bone strength of Cobb**							
Male		Female		Std Error		*p*-value	
120.56 ^a^		94.88 ^b^		5.87		<0.01	

^a^, ^b^ Different superscript letters within a row indicate significant differences (*p* < 0.05).

**Table 4:** Effects of lighting intensity (LI, lux), age, and LI by age interaction on Ross 708 broiler’s back and belly temperature (°C) and bare skin ratio (%).

**Effect of LI on Ross**								
	50 lux	20 lux		5 lux	Average	Std Error		*p*-value
Back skin ratio	19.06	13.32		17.08	16.49	1.18		0.09
Belly temperature	35.19	35.01		34.93	35.04	0.12		0.30
Belly skin ratio	66.87	66.43		67.49	66.93	1.22		0.83
**Effect of age on Ross**								
	Day 28		Day 56		Std Error		*p*-value	
Back skin ratio	31.88 ^a^		1.10 ^b^		1.54		<0.01	
Belly temperature	35.85 ^a^		34.22 ^b^		0.10		<0.01	
Belly skin ratio	66.72		67.13		1.00		0.77	
**LI by age interaction effects of Ross**								
		50 lux		20 lux	5 lux	Std Error		*p*-value
Back temperature	D 28	33.08 ^a^		31.51 ^b^	32.31 ^ab^	0.32		0.03
	D 56	27.00 ^c^		27.06 ^c^	26.70 ^c^			

^a^, ^b^, ^c^ Different superscript letters within a row indicate significant differences (*p* < 0.05). For the LI × age interaction rows, superscript letters reflect comparisons across all six treatment combinations (2 ages × 3 light intensities), rather than comparisons within a single age.

**Table 5:** Effects of lighting intensity (LI, lux), age, and LI by age interaction on Cobb 700 broiler’s back and belly temperature (°C) and bare skin ratio (%).

**Effect of LI on Cobb**									
	50 lux	20 lux		5 lux		Average	Std Error		*p*-value
Back temperature	27.57	27.31		27.74		27.54	1.19		0.28
Back skin ratio	9.38	8.71		10.33		9.47	0.11		0.59
**Effect of age on Cobb**									
	Day 28		Day 56		Std Error			*p*-value	
Back temperature	30.24 ^a^		24.84 ^b^		1.53			<0.01	
Back skin ratio	18.69 ^a^		0.26 ^b^		0.91			<0.01	
**LI by age interaction effects of Cobb**									
		50 lux		20 lux		5 lux	Std Error		*p*-value
Belly temperature	D 28	34.85 ^ab^		34.34 ^bc^		34.82 ^ab^	0.21		<0.01
	D 56	35.23 ^a^		34.99 ^ab^		33.92 ^c^			
Belly skin ratio	D 28	61.03 ^b^		57.97 ^b^		60.79 ^b^	1.83		<0.01
	D 56	76.52 ^a^		73.42 ^a^		64.36 ^b^			

^a^, ^b^, ^c^ Different superscript letters within a row indicate significant differences (*p* < 0.05). For the LI × age interaction rows, superscript letters reflect comparisons across all six treatment combinations (2 ages × 3 light intensities), rather than comparisons within a single age.

**Table 6:** Gait score, footpad condition, and feather cleanliness of Ross 708 broilers under different lighting intensities (LI, lux). All welfare traits were scored using criteria derived from the Welfare Quality^®^ [29] protocol.

Measurement Items	LI of Ross 708					
On day 28	50 lux	20 lux	5 lux	Average	Std Error	*p*-value
Gait score	0.19	0.43	0.31	0.31	0.09	0.18
Footpad	0.67	0.81	0.35	0.61	0.14	0.09
Feather cleanliness	0.80 ^b^	1.07 ^a^	1.00 ^ab^	0.96	0.06	<0.01
On day 56	50 lux	20 lux	5 lux	Average	Std Error	*p*-value
Gait score	1.72 ^b^	2.26 ^a^	2.25 ^a^	2.08	0.12	<0.01
Footpad	1.81	2.38	1.86	2.02	0.18	0.11
Feather cleanliness	2.25 ^b^	2.68 ^a^	2.22 ^b^	2.38	0.11	0.01

^a^, ^b^ Different superscript letters within a row indicate significant differences (*p* < 0.05).

**Table 7:** Gait, footpad condition, and feather cleanliness of Cobb 700 broilers under different lighting intensities (LI, lux). All welfare traits were scored using criteria adapted from the Welfare Quality^®^ [29].

Measurement Items	LI of Cobb 700					
On day 28	50 lux	20 lux	5 lux	Average	Std Error	*p*-value
Gait score	0.50	0.47	0.46	0.48	0.09	0.96
Footpad	1.46	1.71	1.47	1.55	0.19	0.74
Feather cleanliness	0.01	0.05	0.01	0.02	0.03	0.13
On day 56	50 lux	20 lux	5 lux	Average	Std Error	*p*-value
Gait score	2.04 ^b^	2.81 ^a^	2.43 ^ab^	2.43	0.17	<0.01
Footpad	2.46 ^ab^	2.93 ^a^	2.13 ^b^	2.51	0.17	<0.01
Feather cleanliness	1.78	2.03	1.86	1.89	0.09	0.16

^a^, ^b^ Different superscript letters within a row indicate significant differences (*p* < 0.05).

**Table 8:** Assessment of age effects on welfare indicators (gait, footpad dermatitis, feather cleanliness) in Ross 708 and Cobb 700 broilers.

**Measurement Items**	**Ross 708**			
	Day 28	Day 56	Std Error	*p*-value
Gait score	0.31 ^b^	2.08 ^a^	0.06	<0.01
Footpad	0.61 ^a^	2.01 ^b^	0.09	<0.01
Feather cleanliness	0.96 ^b^	2.38 ^a^	0.05	<0.01
**Measurement items**	**Cobb 700**			
	Day 28	Day 56	Std Error	*p*-value
Gait score	0.48 ^b^	2.43 ^a^	0.08	<0.01
Footpad	1.55 ^b^	2.50 ^a^	0.10	<0.01
Feather cleanliness	0.02 ^b^	1.88 ^a^	0.04	<0.01

^a^, ^b^ Different superscript letters within a row indicate significant differences (*p* < 0.05).

## Data Availability

Data are contained within the article.

## Abbreviations

The following abbreviations are used in this manuscript:

LI	Lighting intensity
BW	Body Weight
FCR	Feed conversion ratio
PLF	Precision livestock farming
